# Fourier ptychographic coherence scanning interferometry for 3D morphology of high aspect ratio and composite micro-trenches

**DOI:** 10.1038/s41377-026-02189-6

**Published:** 2026-01-29

**Authors:** Yin Li, Qun Yuan, Xiao Huo, Shumin Wang, Hongtao He, Zhishan Gao

**Affiliations:** 1 School of Electronic and Optical Engineering, Nanjing University of Science and Technology, Nanjing, Jiangsu Province 210094 China; 2 The 13th Research Institute of China Electronics Technology Group Corporation, Shijiazhuang, Hebei Province 050052 China

**Keywords:** Interference microscopy, Imaging and sensing

## Abstract

Non-destructive and accurate characterization of high aspect ratio (HAR) and composite micro-trenches is critical for advanced microfabrication but remains a major challenge. Conventional coherence scanning interferometry (CSI), while widely adopted, suffers from low signal-to-noise ratio (SNR) and limited lateral resolution when applied to HAR and composite microstructures. Here, we present Fourier ptychographic coherence scanning interferometry (FP-CSI), the first transmissive CSI modality that integrates the aperture synthesis strategy of Fourier ptychographic microscopy with the quantitative phase-resolved capability of interferometry. FP-CSI enables robust three-dimensional morphology reconstruction with enhanced SNR and improved lateral resolution, without reliance on iterative phase retrieval. We demonstrate accurate measurements of a HAR micro-trench (300 μm depth, 30:1 aspect ratio) and micro-electro-mechanical system (MEMS) devices (aspect ratios 6:1–20:1). FP-CSI achieves lateral resolution up to the incoherent diffraction limit and maintains this performance even at trench bottoms. Owing to its fidelity, robustness, and non-destructive operation, FP-CSI provides a powerful new metrology platform for next-generation semiconductor inspection, precision manufacturing, and emerging micro-optoelectronic systems.

## Abstract (web summary)

A transmissive Fourier ptychographic coherence scanning interferometry method provides accurate 3D morphology measurements of high aspect ratio and composite micro-trenches, enhancing both the signal-to-noise ratio and lateral resolution.

## Graphical abstract

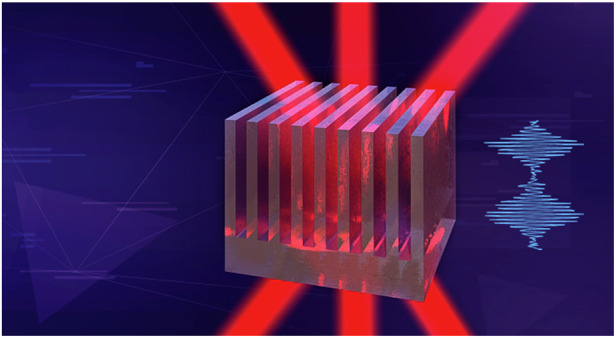

## Introduction

High aspect ratio (HAR) micro-trenches, typically characterized by narrow width and significant depth, attach critical importance to advanced manufacturing^1–3^. For example, in micro-electro-mechanical systems (MEMS)^4^, GaN-based light-emitting diodes^5^, and meta-optics^6^, many micro-nano devices require HAR micro-trenches not only to facilitate the integration and miniaturization of electronic components but also to provide an enlarged response area for performance and efficiency improvement. In addition, the three-dimensional (3D) morphology of these structures has a significant impact on their exceptional performance, making accurate profiling essential for inline process monitoring and dimensional control in precision microfabrication workflows. Consequently, scanning electron microscopy (SEM)^7–9^ has become widely utilized in industrial institutions. Nonetheless, cross-sectional measurement inevitably comes with specimen damage, thus limiting its suitability for inline or repeatable inspection. This limitation has motivated the development of non-destructive optical metrology techniques^10–13^, including spectroscopic reflectometry^14,15^, through-focus scanning optical microscopy^16,17^, optical scatterometry^18,19^, optical coherence tomography^20^, and coherence scanning interferometry (CSI)^21–23^. Among these, CSI is probably the most promising technique for non-destructive 3D metrology thanks to its capability of recovering the topography of microstructures rather than just statistical values (width and depth). Its broadband low-coherence illumination further enhances its effectiveness as a quantitative phase recovery tool, tackling the issue of phase ambiguity^24^. Moreover, CSI’s high numerical aperture (NA) objective lenses and high-precision scanning module facilitate high-resolution 3D topographic reconstruction.

Despite all the advantages, CSI still faces critical challenges in the metrology of HAR microstructures. Specifically, high-NA illumination interacting with deep or steep features leads to multiple internal scattering, severe edge diffraction, and complex light field modulation. These effects collectively degrade the interference contrast and reduce the signal-to-noise ratio (SNR), thereby limiting the measurement fidelity. To tackle these challenges, Jo et al.^25^ developed a low-NA white-light CSI method aimed at preventing multiple modulation effects, which enables the measurements of through-silicon vias with aspect ratios up to 11.2. However, this approach’s significantly reduced lateral resolution (~6 μm) restricts its applicability to fine spatial discrimination. Ma et al.^23^ proposed an aberration-compensated CSI technique that utilizes a deformable mirror to correct for sample-induced wavefront distortions. This technique facilitates high-NA imaging and allows the accurate measurement of a single micro-trench with aspect ratios up to 20:1. Nevertheless, it is constrained by the correction range of the deformable mirror, rendering it ineffective for complex structures. This inefficiency arises because spatially varying aberrations require multiple iterative compensations. Qiao et al.^22^ introduced a deconvolution-based CSI method that enhances interference contrast by matching measured data with a theoretical 3D point spread function model. While this method has proven effective in certain situations, it faces challenges due to the potential similarities among numerical models of composite microstructures.

Although the above methods have demonstrated the feasibility of applying CSI to HAR microstructure measurement, they collectively expose a fundamental trade-off: low-NA configurations enhance SNR and interference contrast but fail to resolve structural details, while high-NA systems offer better lateral resolution at the expense of signal degradation due to complex light-matter interactions. As a result, achieving robust, high-resolution 3D reconstruction of HAR and composite microstructures remains an unresolved challenge in optical metrology. Fourier ptychographic microscopy (FPM)^26–30^, a non-interferometric imaging strategy, offers a promising framework to overcome this limitation of conventional CSI. By synthetically expanding spatial-frequency coverage through angularly varied illumination, FPM enables high-resolution reconstruction from low-NA acquisitions and mitigates the trade-off between resolution and field of view^31–34^. This strategy is typically implemented in a transmissive configuration, which naturally reduces sample-induced modulation, an issue that often limits signal quality in reflective CSI, particularly when imaging deep or steep features. Meanwhile, FPM relies on complex iterative algorithms to retrieve phase information from intensity-only measurements, which can be computationally intensive and sensitive to noise^35–38^. In contrast, CSI offers direct and quantitative phase acquisition with high axial accuracy but suffers from the SNR-to-resolution trade-off inherent in reflective setups. By integrating the transmissive angular spectrum fusion of FPM with the phase-resolved capability of CSI, a hybrid approach can be established that combines the complementary strengths of FPM and CSI. This fusion approach not only mitigates the degradation in signal quality observed in reflective CSI but also eliminates the need for iterative phase retrieval typical of FPM. As a result, this framework enables robust, high-resolution 3D reconstruction of HAR and composite microstructures, offering a compelling solution to long-standing challenges in optical metrology.

In this paper, we present Fourier ptychographic coherence scanning interferometry (FP-CSI), to our knowledge, the first transmissive CSI modality based on angular spectrum scanning. Designed for robust and high-precision 3D metrology of HAR micro-trenches, FP-CSI employs a transmissive near-infrared Linnik interferometer in which the sample is illuminated by a series of quasi-plane waves at variable incident angles. This configuration mitigates optical field modulation in HAR features and enhances both SNR and interference contrast. The resulting angularly scanned interferograms are converted into phase maps and fused in the Fourier domain via sub-aperture stitching, enabling high-resolution topography reconstruction with lateral resolution comparable to that of a high-NA system. Compared with traditional CSI, FP-CSI overcomes the fundamental trade-off between lateral resolution and signal quality in measurements of HAR microstructures. Moreover, the intrinsic quantitative phase acquisition and strict optical conjugation of CSI eliminate the need for the iterative phase retrieval required in conventional FPM. The method is validated on HAR micro-trenches and multilayer MEMS devices. We further demonstrate lateral resolution at the incoherent diffraction limit on a resolution target and sustained resolving capability even at the bottoms of simulated trenches with aspect ratios exceeding 10:1. In summary, FP-CSI combines the quantitative phase accuracy of interferometry with the aperture synthesis principle of Fourier ptychography, overcoming the long-standing SNR-to-resolution trade-off in HAR microstructure metrology and establishing a versatile platform for advanced microfabrication inspection.

## Results

### Optical setup

**Fig. 1:**
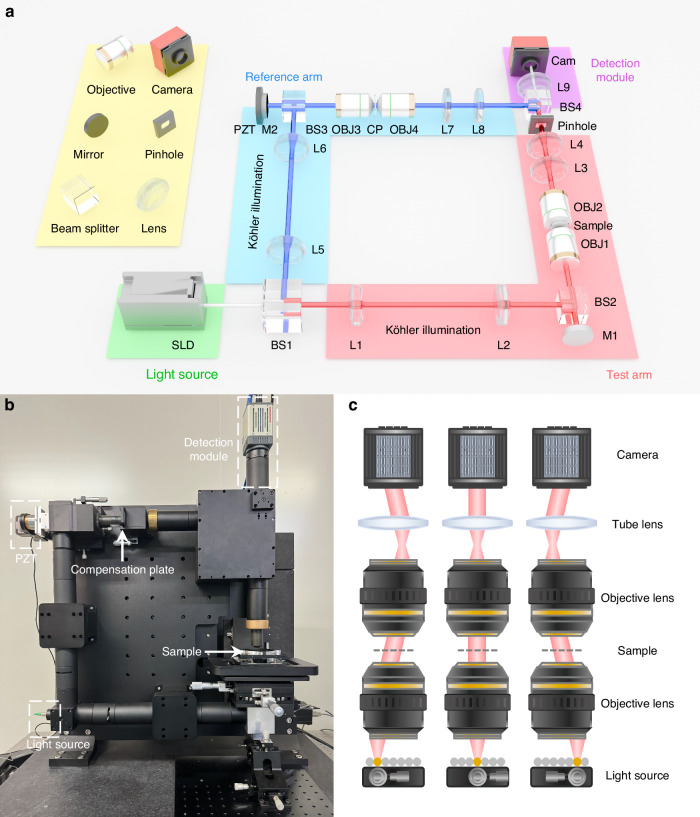
Configuration and working principle of the FP-CSI system. **a** Optical schematic of the FP-CSI framework based on a transmissive Linnik interferometer. A near-infrared superluminescent diode (SLD) provides quasi-point illumination at various positions to generate angularly diverse illumination. The reference and test arms are symmetrically configured in a Linnik arrangement with Köhler illumination (L1, L2, L5, L6), near-infrared objectives (OBJ1–4), and relay lenses (L3, L4, L7, L8). In the test arm, a pinhole is conjugated with the objective pupil. In the reference arm, a piezoelectric transducer (PZT) modulates the optical path during axial scanning. Additionally, a compensating plate (CP) with the same thickness and material as the sample is positioned in the parfocal plane of OBJ3 and OBJ4. Interferograms are relayed by a tube lens (L9) and captured by a near-infrared InGaAs camera (Cam), which functions as the detection module. SLD superluminescent diode, BS beam splitter, L lens, M mirror, OBJ objective lens, CP compensation plate, Cam near-infrared camera, PZT piezoelectric transducer. **b** Setup of the experimental FP-CSI system corresponding to the schematic in (**a**). **c** Conceptual illustration of angularly varying quasi-parallel illumination generated by translation of the fiber source. Larger source displacements correspond to increased incident angles and the sampling of higher spatial-frequency components within the sample’s angular spectrum

Figure 1a shows the schematic of the proposed FP-CSI system, and Fig. 1b presents the experimental implementation. The system employs a near-infrared superluminescent diode (Thorlabs SLD1325, center wavelength 1325 nm, bandwidth 100 nm) as the illumination source. Mounted on a motorized XY translation stage (Thorlabs M30XY), the source provides quasi-point illumination at different positions across the illumination plane. Both the test and reference arms adopt a symmetric transmissive Linnik configuration, each incorporating a Köhler illumination module, a pair of custom-designed near-infrared objectives (20×, NA = 0.5), and a relay lens system. The test arm contains a pinhole (JCOPTIX MPS1-1000, 1 mm diameter) conjugated with the objective pupil (i.e., the objective’s back focal plane) to suppress spurious diffraction components, while the reference arm integrates a piezo actuator (PI P-737.5SL) for precise optical-path modulation during axial scanning. The resulting interferograms are imaged onto the detector plane through the tube lens and captured by a near-infrared InGaAs camera (Hamamatsu C12741-03).

The Linnik arrangement supports high-NA objectives while maintaining sufficient working distance, ensuring both resolution and compatibility with a wide range of microstructures and substrates. The sample surface is positioned at the common focal plane of the objectives in the test arm and mounted on a high-precision translation stage (NanoMotions PFR150-400-U-S) for vertical scanning. A compensating plate of identical thickness and material to the sample substrate is placed in the reference arm to correct for chromatic dispersion. As illustrated in Fig. 1c, an off-axis quasi-point source generates a quasi-parallel beam incident on the sample at an oblique angle $\theta$. Larger source displacements correspond to greater incident angles, thereby encoding variable spatial-frequency components of the sample. This configuration enables angular spectrum scanning and quantitative phase acquisition, forming the hardware foundation for robust, high-resolution 3D topographic reconstruction of HAR and composite micro-trenches.

### Experimental validation of the transmissive configuration

An essential innovation of FP-CSI is the adoption of a transmissive interferometric architecture, which offers significant advantages over traditional, reflective CSI systems. In reflective systems, light interacts with the sample twice. The double interaction leads to excessive modulation and signal distortion, which severely degrades the SNR and fringe visibility. This degradation is particularly problematic in HAR microstructures, where internal scattering and edge diffraction pose additional challenges. In contrast, transmissive quasi-parallel illumination enables light to pass through the sample only once at a small divergence angle, thereby reducing cumulative modulation and enhancing both the SNR and interference contrast.

**Fig. 2:**
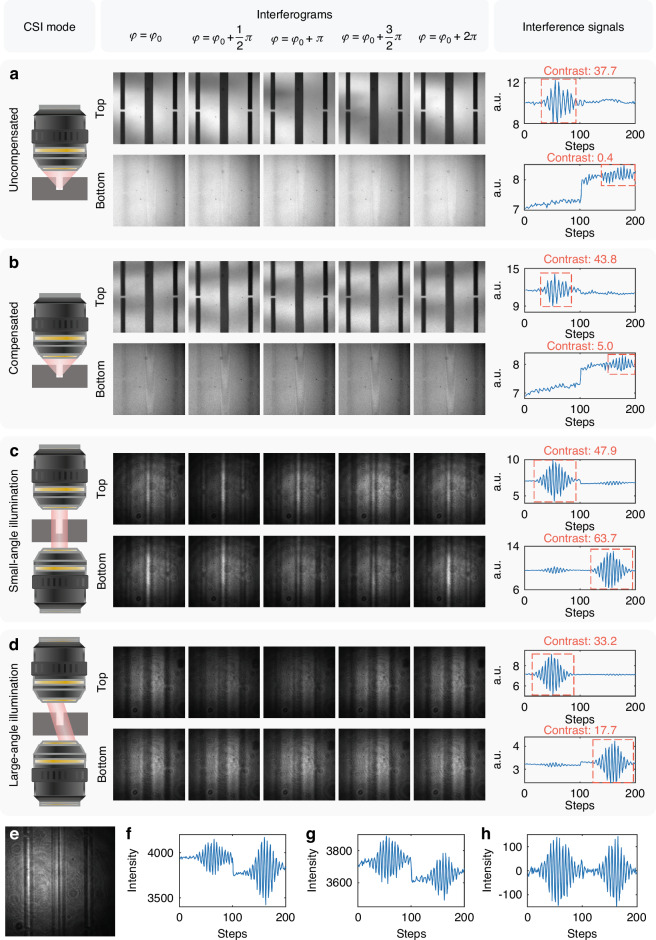
Comparative interferometric measurements of a HAR micro-trench (30 μm width, 300 μm depth) using reflective Linnik and FP-CSI configurations. **a**–**d** Representative interferograms at selected axial positions (step size $\Delta \varphi =\pi /2$) and corresponding interference signals. Reflective Linnik interferometric signals without (**a**) and with (**b**) deformable-mirror-based wavefront compensation. FP-CSI interferometric signals acquired under normal incidence (**c** $\theta =0^\circ$, NA = 0) and oblique incidence (**d** $\theta =15.8^\circ$, NA = 0.27). **e** FP-CSI interferogram obtained without pinhole filtering, exhibiting prominent diffraction patterns and reduced contrast, in contrast to the high-contrast interference fringes in (**c**) and (**d**) obtained with the pinhole in place. Raw interference signals extracted from the trench bottom at $\theta =8.6^\circ$ (**f**, NA = 0.15) and $\theta =23.5^\circ$ (**g**, NA = 0.40). **h** Corrected interference signal obtained from (**g**) after empirical mode decomposition

To experimentally validate the superiority of the transmissive framework, we compared measurements of a HAR micro-trench (width: 30 μm, depth: 300 μm) acquired with state-of-the-art reflective CSI^23^ and FP-CSI. As shown in Fig. 2a, the reflective Linnik system displayed high fringe contrast at the top surface (Contrast: 37.7) but almost no detectable signal from the trench bottom (Contrast: 0.4), owing to accumulated wavefront distortion and scattering. Incorporating deformable-mirror compensation (Fig. 2b) modestly improved the bottom signal (Contrast: 5.0) and slightly enhanced the top surface (Contrast: 43.8), but overall gains were limited. In contrast, FP-CSI provided substantially higher signal quality at both surfaces. Under normal incidence (Fig. 2c, $\theta =0^\circ$, NA = 0), contrast reached 47.9 (top) and 63.7 (bottom). Even under oblique illumination (Fig. 2d, $\theta =15.8^\circ$, NA = 0.27), the bottom contrast remained 17.7, far exceeding that of the compensated reflective system, while the top surface contrast remained acceptable at 33.2. These results demonstrate that, although adaptive optics can partially mitigate the signal degradation of reflective CSI, FP-CSI achieves an intrinsically higher SNR, especially at the trench bottom. The definition and calculation of interference contrast are provided in Supplementary Note S2.

Despite this improvement, HAR microstructures still induce edge diffraction that reduces the fringe visibility. To suppress such artifacts, a pinhole was inserted at the plane optically conjugate to the objective pupil in the test arm, acting as a spatial filter that removes high-frequency diffraction components and preserves the essential angular spectrum. With the pinhole in place, Fig. 2c, d demonstrates significantly improved contrast, whereas the interferograms obtained without the pinhole (Fig. 2e) exhibit severe spurious patterns. The pinhole diameter was selected based on the projected size of the quasi-point source at the pupil plane, and its position was dynamically adjusted during angular scanning to remain conjugate with the illumination source, analogous to the aperture-scanning strategy in Fourier ptychography^39,40^. This configuration not only suppresses diffraction noise but also establishes a stable frequency domain reference that facilitates accurate sub-aperture stitching.

However, even after the diffraction artifacts are suppressed, nonnegligible signal distortions remain under large-angle illumination. These issues arise primarily from projection-induced asymmetry under oblique illumination and intensity drift in the interference envelope, particularly near sidewalls where grazing incident light leads to uneven optical-path contributions. As shown in Fig. 2f ($\theta =8.6^\circ$, NA = 0.15) and Fig. 2g ($\theta =23.5^\circ$, NA = 0.40), the interference signals at larger angles exhibit reduced peak amplitude, asymmetric envelope profiles, and baseline fluctuations that hinder reliable phase demodulation. These distortions become increasingly pronounced with greater illumination angles, rendering correction indispensable. To address this, we applied empirical mode decomposition^41^ to adaptively remove the envelope drift and restore the signal quality. The corrected signal (Fig. 2h) exhibits improved symmetry and stability, significantly enhancing the robustness of phase extraction. This correction is crucial for accurate morphology recovery of HAR microstructures under challenging large-angle illumination.

### The FP-CSI reconstruction algorithm

**Fig. 3:**
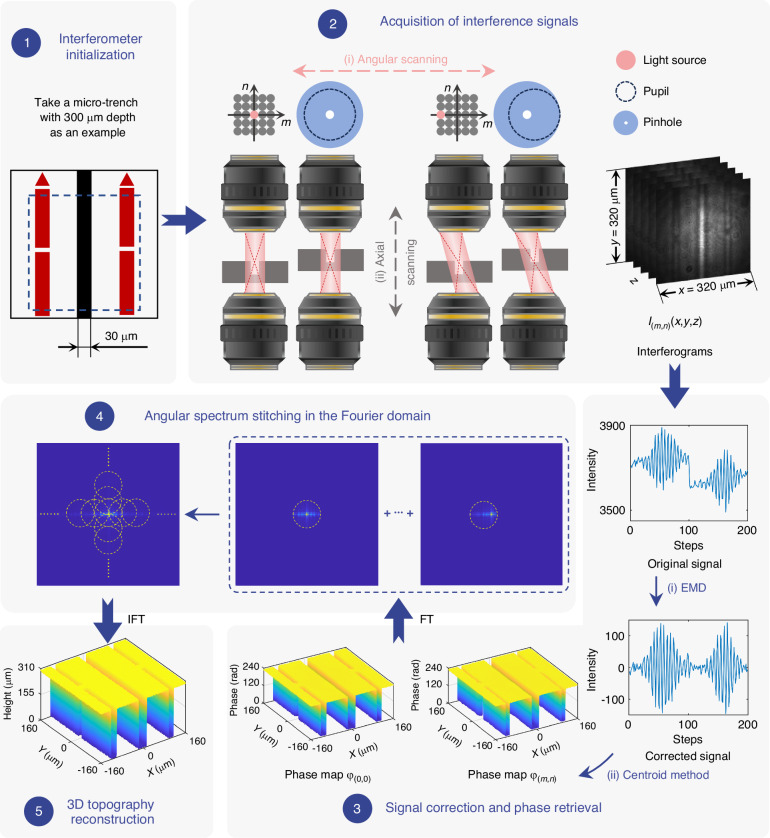
Reconstruction workflow of the FP-CSI algorithm. The workflow is illustrated using a HAR micro-trench example with a width of 30 μm and a depth of 300 μm. Step 1: Interferometer initialization. The top surface of the sample is located at the parfocal plane of the test-arm objectives. Meanwhile, a compensation plate with identical thickness and material to the sample is placed in the reference arm. Step 2: Axial and angular scanning to acquire interferograms. This step provides depth-resolved signals across multiple incidence angles. Step 3: Signal correction using empirical mode decomposition and phase extraction via the centroid method. Empirical mode decomposition is applied to the interferometric signals, especially at large angles, to suppress low-frequency intensity drift and envelope asymmetry. The centroid method is then employed to extract high-fidelity phase maps for each angular view. Step 4: Angular spectrum stitching in the Fourier domain. Each angularly resolved phase map is assigned to its corresponding subregion in the spatial-frequency domain, and all subregions are linearly combined in the Fourier domain to reconstruct the complete phase distribution. Step 5: Conversion of the stitched phase into 3D topography. The stitched phase is converted into surface height, resulting in a high-resolution 3D topography of the sample. EMD empirical mode decomposition, FT Fourier transform, IFT inverse Fourier transform

To recover high-resolution 3D morphology from the interference signals acquired under varying spatial frequencies, FP-CSI extends the aperture synthesis principle of FPM to CSI. Figure 3 illustrates the reconstruction workflow, demonstrated on a micro-trench with a 30 μm width and 300 μm depth. The process begins with interferometer initialization, in which the sample’s top surface is positioned at the parfocal plane of the test-arm objectives, and a compensation plate is inserted to correct chromatic dispersion, ensuring optical-path match and stable interference. The reconstruction algorithm comprises three main stages:

1. **Acquisition of phase maps**
  Interferograms are recorded through combined axial and angular scanning.
  **Axial scanning**
    The reflective mirror in the reference arm is translated in increments of λ/8 to achieve a stepwise optical-path difference modulation of λ/4, where λ denotes the illumination center wavelength. To ensure that the sample plane coincides with the optimal interference position throughout the axial scanning, the sample is synchronously translated by
    1 $$\Delta {z}_{\mathrm{sample}}=\frac{1}{4}\lambda \cdot \frac{1}{n-{n}_{0}}$$
    where $n$ and ${n}_{0}$ are the refractive indices of the sample and air, respectively.
  **Angular scanning**
    Angular diversity is introduced by shifting the quasi-point source across the illumination plane. The maximum illumination angle is limited by the objective’s NA. To maintain spatial conjugation, the pinhole at the pupil plane is co-shifted with the light source during scanning. The oblique angle of the incident light is
    2 $$\theta (m,n)=\arctan \frac{\beta \sqrt{{m}^{2}+{n}^{2}}}{f}$$
    where $(m,n)$ is the spatial coordinates of the translating quasi-point light source, $\beta$ is the optical magnification that images the light source onto the objective pupil plane, and $f$ represents the focal length of the objective lens. Therefore, each set of angular and axial scanning positions corresponds to an interferogram for subsequent phase extraction.
  **Signal correction and phase extraction**
    Interference signals acquired at large angles often suffer from intensity drift and asymmetrical envelopes due to projection effects. To address this, empirical mode decomposition is applied to adaptively suppress low-frequency distortions. The centroid method^42^ is then used to extract high-fidelity phase maps for each angular view.

(2) **Angular spectrum stitching in the Fourier domain**
  Each angularly resolved phase map ${\varphi }_{(m,n)}(x,y)$ at object coordinates $(x,y)$ is associated with a specific subregion of the spatial-frequency domain. The effective frequency component is
  3 $${O}_{(m,n)}(u,v)= {\mathcal F} \{{\varphi }_{(m,n)}(x,y)\}\cdot {P}_{(m,n)}(u,v)$$
  where ${\mathcal F}$ denotes the Fourier transform and ${P}_{(m,n)}(u,v)$ is the pupil function,
  4 $${P}_{(m,n)}(u,v)=\left\{\begin{array}{l}1,\,\,{(u-\beta m)}^{2}+{(v-\beta n)}^{2}\le {r}^{2}\,\\ 0,\,\,{(u-\beta m)}^{2}+{(v-\beta n)}^{2} > {r}^{2}\end{array}\right.$$
  with pupil coordinates $(u,v)$ and spot radius $r$ of fiber source image in the pupil plane. The radius determines the effective cutoff frequency, thereby suppressing high-frequency noise. The complete phase distribution is reconstructed by linearly combining all subregions in the Fourier domain:
  5 $$\varphi (x,y)={ {\mathcal F} }^{-1}\{\mathop{\sum }\limits_{m}\mathop{\sum }\limits_{n}{O}_{(m,n)}(u,v)\}$$
  No iterative procedure is required, since adjacent subregions overlap sufficiently. Minor inconsistencies within overlapping regions are resolved by averaging, as detailed in Supplementary Note S4. This overlap strategy enhances computational efficiency and has demonstrated reliable performance in practical experiments.
(3) **3D topographic reconstruction**
  The final stitched phase map is converted to surface height to produce a high-resolution 3D topography of the sample. Detailed experimental parameters, including the number of angular views and hardware control settings, are provided in the “Imaging acquisition and analysis” section. The forward model of transmissive CSI, the specific reconstruction algorithm, and the measurement results for the representative sample (30 μm width, 300 μm depth) are presented in Supplementary Notes S1, S2, and S3, respectively.

### Topographic reconstruction of HAR micro-trenches

**Fig. 4:**
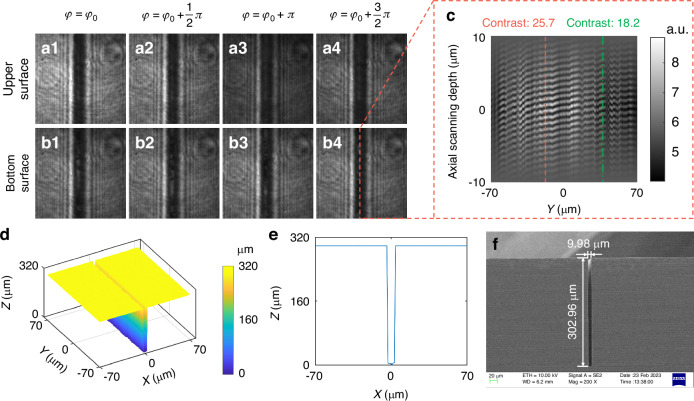
Topographic reconstruction of a HAR micro-trench (10 μm width, 300 μm depth) using FP-CSI. **a1**–**a4** Interferograms of the trench upper surface. **b1**–**b4** Interferograms of the trench-bottom surface. **c** Axial interference signal extracted along the sampling line indicated in (**b4**). The high-contrast axial interference fringes across the scanning range confirm stable phase retrieval from the trench bottom, while local variations in fringe contrast suggest surface roughness or scattering features. **d** Reconstructed 3D topography. **e** Cross-sectional profile extracted from (**d**). **f** SEM image of the same micro-trench for validation

To validate the performance of FP-CSI in reconstructing HAR features, we measured a micro-trench with a width of 10 μm and a depth of 300 μm. Figure 4a1–a4 and b1–b4 display the interferograms acquired from the top and bottom surfaces of the trench, respectively. Despite the extreme aspect ratio and narrow width, the interference patterns of both surfaces maintain high fringe visibility, confirming the system’s ability to capture coherent signals at depth. Figure 4c shows the depth-resolved interference signal extracted along the sampling line marked in Fig. 4b4. The high-contrast coherence envelope across scanning positions further demonstrates the system’s robustness in resolving deep surfaces. Variations in fringe contrast at the trench bottom indicate the presence of surface roughness or scattering features.

Figure 4d presents the reconstructed 3D topography of the micro-trench, with a representative cross-sectional profile extracted in Fig. 4e. For validation, an SEM image with nanometer accuracy of the same trench (Fig. 4f) reveals a symmetric, arc-shaped bottom contour in excellent consistency with the FP-CSI reconstruction. Quantitative assessment was performed by measuring characteristic parameters of the micro-trench across ten FP-CSI trials, following the area method defined in ISO/DIS 25178-700:2022^43^. The average width and depth relative error from SEM measurements are only 1.00% and 0.24%, with repeatability of 0.95% and 0.59%, respectively (Supplementary Table S2). These results confirm that FP-CSI achieves high accuracy and repeatability in reconstructing HAR microstructures, establishing its suitability for metrology applications.

### Topographic reconstruction of MEMS multilayer trench devices

**Fig. 5:**
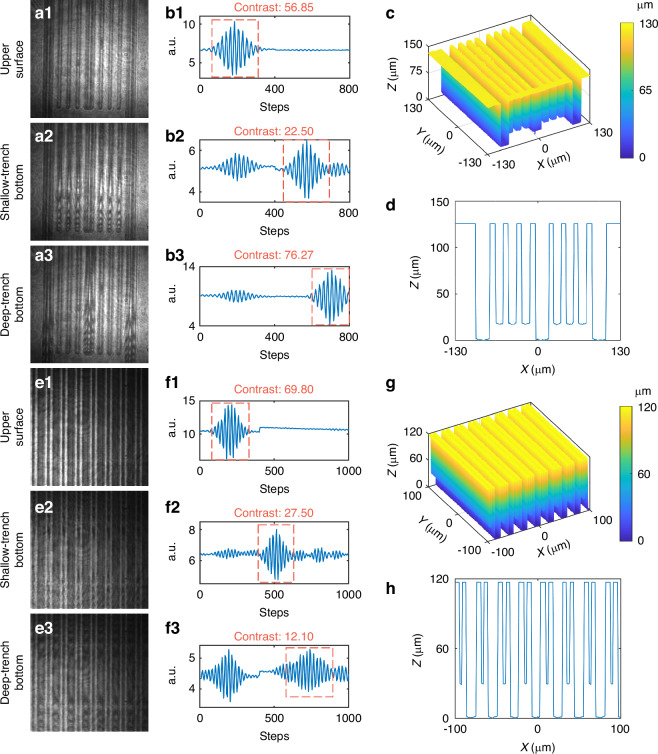
Topographic reconstruction of two MEMS pressure sensors with multilayer trench structures using FP-CSI. **a1**–**a3** Interferograms from pressure sensor 1 at the top surface, the bottom of the shallow trench, and the bottom of the deep trench, respectively. **b1**–**b3** Corresponding axial interference signals from representative points in pressure sensor 1. **c** Reconstructed 3D topography of pressure sensor 1. **d** Cross-sectional profile extracted from (**c**). **e1**–**e3** Interferograms of pressure sensor 2, showing three distinct structural planes associated with different trench levels of the multilayer pressure sensor. **f1**–**f3** Axial interference signals from representative locations in pressure sensor 2, corresponding to the three structural planes. **g** Reconstructed 3D topography of pressure sensor 2. **h** Cross-sectional profile extracted from (**g**)

To further demonstrate the versatility of FP-CSI for complex microfabricated devices, we applied it to the characterization of two MEMS pressure sensors with multilayer comb micro-trenches. Figure 5a1–a3 and e1–e3 show interferograms acquired from the top surface, the bottom surface of the shallow trench, and the bottom surface of the deep trench, respectively. High-contrast fringes are observed across all structural layers, indicating that coherent signals are preserved even within multilayer microstructures. The corresponding axial interference signals (Fig. 5b1–b3 and f1–f3) exhibit distinct coherence peaks at each depth, confirming the system’s ability to resolve stratified features within composite microstructures.

The reconstructed 3D morphologies of the two MEMS devices are presented in Fig. 5c, g, with representative cross-sectional profiles shown in Fig. 5d, h. The reconstructions reveal sharp delineation of multiple trench levels and exhibit high structural fidelity when compared with SEM images (Supplementary Fig. S2e–f). Quantitative validation was carried out using the same area-based evaluation method. Across ten repeated FP-CSI measurements for each device, the average width and depth relative error from SEM were below 0.71%, with repeatability under 0.97% (Supplementary Tables S3 and S4). These results establish that FP-CSI provides accurate, repeatable, and non-destructive characterization of multilayer MEMS trench structures, underscoring its potential as a robust metrology tool for composite microstructures in industrial applications.

### Quantitative lateral resolution characterization in HAR micro-trenches

**Fig. 6:**
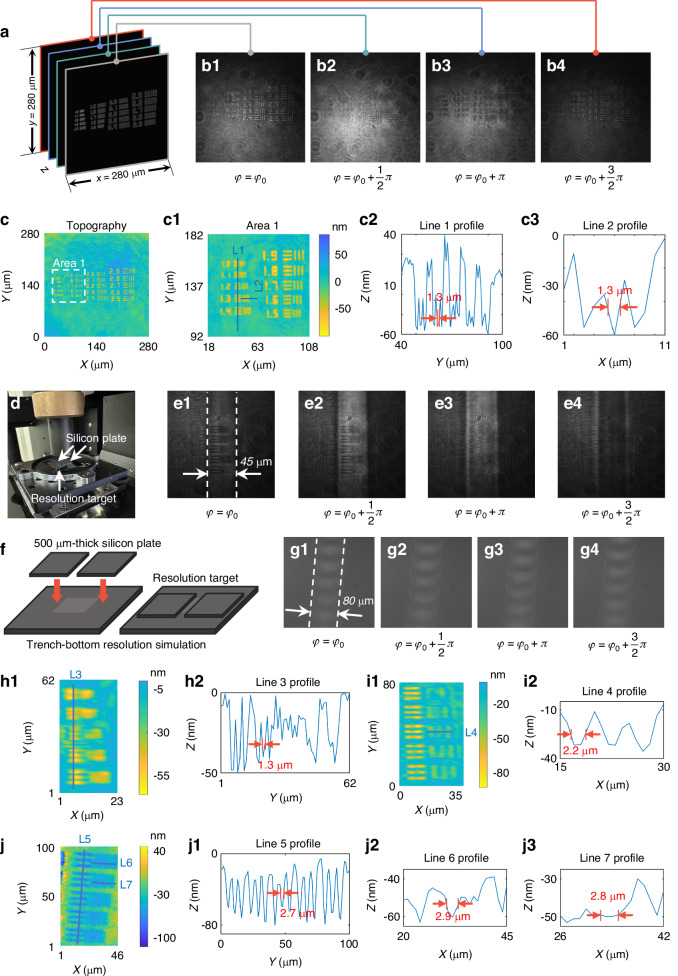
Quantitative evaluation of the lateral resolution and trench-bottom imaging of FP-CSI. **a** Custom phase-only resolution target consisting of line pairs with varying spatial frequencies. **b1**–**b4** Representative FP-CSI interferograms of the bare resolution target. **c** Reconstructed morphology of the bare resolution target. **c1** Reconstructed topography of a selected subregion (area 1 in **c**). **c2**, **c3** Cross-sectional profiles of line pairs (Line 1, Line 2) in (**c1**). **d**, **f** Equivalent HAR trench structure formed by stacking two intrinsic silicon wafers on the resolution target, placing the line pairs at the trench bottom. **e1**–**e4** Representative FP-CSI interferograms of the resolution target in the stacked-wafer configuration (as in **d**, **f**). **g1**–**g4** Interferograms acquired with a reflective Linnik interferometer. **h1**, **i1** FP-CSI reconstructions of line pairs at the trench bottom (aspect ratio ~11:1). **h2**, **i2** Cross-sectional profiles from (**h1**, **i1**). **j** Reflective Linnik reconstruction of an 80-μm-wide trench (aspect ratio 6.25:1). **j1**–**j3** Corresponding cross-sections from (**j**)

To quantitatively evaluate the lateral resolution of FP-CSI, a custom phase-only resolution target is fabricated, consisting of line pairs with gradually varying spatial frequencies (Fig. 6a). Representative interferograms acquired from FP-CSI are displayed in Fig. 6b1–b4, demonstrating high fringe visibility across the line-pair regions. By stitching in the spatial-frequency domain, the morphology of the bare resolution target was reconstructed (Fig. 6c), with selected subregions (area 1, Fig. 6c1) and cross-sectional profiles (Fig. 6c2, c3). Line pairs with a spacing of 1.3 μm are clearly resolved, and no significant anisotropy was observed between *x*- and *y*-oriented line pairs. This resolution matches the theoretical incoherent diffraction limit, $\lambda /(2NA)$ = 1.3 μm ($\lambda$=1.3 μm, NA = 0.5), confirming that FP-CSI achieves its intrinsic system resolution.

To our knowledge, no prior experimental research has quantitatively assessed the lateral resolution at the bottom of HAR micro-trenches. To investigate this, we constructed an equivalent trench by stacking two 500-μm-thick intrinsic silicon wafers on the resolution target, creating a 45-μm-wide groove with an aspect ratio of approximately 11:1 (Fig. 6d, f). This setup positioned the line pairs at the bottom of the trench and provided a rigorous test of the performance of FP-CSI in trench-bottom imaging. Representative interferograms obtained with FP-CSI (Fig. 6e1–e4) and the reconstructed topographies indicate that the resolution along the trench axis (*y*-direction, Fig. 6h1, h2) remains largely unchanged, successfully resolving features down to 1.3 μm. In contrast, the effective resolution modestly decreases to about 2.2 μm across the trench (*x*-direction, Fig. 6i1, i2). This reduction is primarily attributed to diffraction and stray light resulting from the sharp edges of the wafers. Additionally, the sensor pixel size (20 μm) and phase-retrieval errors further restrict the effective resolution.

For comparison, the trench-bottom experiment was repeated using the state-of-the-art reflective Linnik interferometer^23^ with identical objective lenses. In the reflective arrangement, the inherent increase in optical modulation dramatically reduces fringe visibility at the bottom. Even with a trench width of 80 μm (aspect ratio 6.25:1), the interference contrast was low (Fig. 6g1–g4), and further reduction of the trench width rendered the signal insufficient for reliable demodulation. When the trench width was set to 45 μm, corresponding to the parameter used in FP-CSI (aspect ratio 11:1), the interference fringes became too weak to recover. Therefore, the reconstructed topography at 80 μm width (Fig. 6j) and corresponding cross-sectional profiles (Fig. 6j1–j3) are presented. These results reveal partial preservation of resolution along the *y*-direction but a degraded resolution of ~2.9 μm along the *x*-direction, significantly worse than FP-CSI. This comparison highlights the advantage of the transmissive configuration, which maintains high-resolution imaging even at considerable depths within HAR microstructures.

Notably, increasing the illumination spatial frequency (i.e., larger incident angles) can further enhance resolution for vertical line pairs at the trench bottom. However, in the present implementation, phase retrieval is partially limited by system noise and stray light. With optimized demodulation algorithms, improved stray-light suppression, and finer detector sampling, the *x*-direction resolution could be further improved to better than the current 2.2 μm.

In summary, FP-CSI achieves a quantitative lateral resolution up to the incoherent diffraction limit in the bare resolution target and preserves near diffraction-limited performance at the bottom of trenches with aspect ratios exceeding 10:1. Compared with reflective interferometry, FP-CSI preserves high-resolution imaging in challenging HAR conditions, owing to its angular spectrum synthesis in a transmissive configuration. These findings establish FP-CSI as a powerful tool for non-destructive metrology of HAR microstructures and, more broadly, for volumetric imaging of thick, scattering samples.

## Discussion

### Advantages of FP-CSI for non-destructive measurement

By integrating angular spectrum synthesis with low-coherence interferometric imaging, FP-CSI addresses critical limitations of both conventional CSI and FPM. Unlike reflective interferometric architectures, its transmissive configuration minimizes sample-induced optical modulation, thereby preserving the SNR and enhancing interference contrast. This enhancement is particularly valuable for characterizing HAR microstructures such as deep trenches, vertical vias, and multilayer MEMS devices. Angular spectrum scanning further alleviates the trade-off between lateral resolution and signal quality inherent to CSI. Multiple illumination angles provide dense spatial-frequency coverage and enable high-resolution reconstructions without sacrificing fringe visibility. Compared with FPM, which depends on intensity-only measurements and iterative phase retrieval, FP-CSI directly recovers quantitative phase maps from interferograms, avoiding convergence instability and reducing computational overhead. Collectively, these features position FP-CSI as a powerful non-destructive solution for high-resolution metrology of HAR microstructures, extending quantitative measurement capabilities to thick, transmissive samples.

A more detailed comparison of the proposed FP-CSI method with current state-of-the-art techniques is provided in Supplementary Note S5, which offers key performance metrics for each method, including measurable quantities, achievable aspect ratios, acquisition time, and major limitations. In summary, FP-CSI has introduced innovative methods for achieving high-resolution 3D reconstruction, applicable not only to microstructures with an aspect ratio of up to 30:1 but also to composite MEMS devices. This advancement overcomes the measurement limitations inherent in current morphology metrology techniques.

### System calibration and temporal optimization

Accurate reconstruction relies on precise calibration of angular illumination. Each illumination angle corresponds to a spatial-frequency subregion determined by the light source position in the conjugate plane of the objective pupil. In the present system, mechanical scanning with a two-dimensional stage enables angular diversity, and a pupil-plane camera provides real-time monitoring and feedback to correct positional errors. This scheme ensures robust frequency domain registration and stable 3D topographic reconstructions. As near-infrared implementations advance, alternative technologies, such as digital micromirror devices^44^, can substitute for mechanical motion. This substitution will effectively eliminate calibration overhead and reduce the time required for angular-scanning acquisition.

Temporal performance of FP-CSI can be further improved by increasing acquisition efficiency, reducing sampling, and accelerating computation. Firstly, efficiency gains arise from faster illumination steering, higher-frame-rate sensors, and synchronized parallel triggering of the illumination, axial actuator, and camera. Additionally, sampling can be reduced by integrating established FPM accelerations, such as source-coded or multiplexed illumination^45,46^, thereby requiring fewer interferograms for a target resolution. Moreover, on the computational side, GPU-accelerated reconstruction pipelines and deep learning algorithms for signal correction and phase prediction are expected to reduce processing time and enhance system automation. Collectively, these hardware, control, and algorithmic measures enable FP-CSI to scale toward large-area, high-efficiency metrology and dynamic, real-time measurements.

### Scalability and outlook of FP-CSI

The FP-CSI technique functions in transmission mode at the probing wavelength, necessitating that samples be transparent. Consequently, FP-CSI is especially suitable for quality control before metallization. The current implementation of FP-CSI is optimized for characterizing microstructures that exhibit sparse or axis-aligned spatial-frequency content, such as HAR micro-trenches. By aligning the angular scanning along the principal frequency axes (the *x*- and *y*-directions), this method efficiently captures essential frequency components while minimizing data redundancy and computation overhead. This targeted sampling strategy has been experimentally validated, resulting in high-fidelity 3D topographic reconstructions of both the tops and bottoms of micro-trenches, demonstrating excellent agreement with SEM.

In principle, FP-CSI can be extended to more complex morphologies, including through vias and curved or multilayer structures, where spatial-frequency content becomes denser and less directional. Achieving high-precision reconstructions of these features necessitates broader angular coverage and denser sampling to ensure sufficient filling of the spatial-frequency domain. Such capabilities will expand the technique’s applicability to more composite semiconductor devices and potentially to thick biological samples.

In addition to morphological complexity, the FP-CSI forward model can extend beyond homogeneous microstructures to multilayer and heterogeneous substrates by incorporating optical-path terms that correspond to interfaces and combining envelope separation strategies derived from CSI^47–49^. This capability enables quantitative topography of microstructures fabricated on complex substrates typical of advanced MEMS and integrated photonics.

### Conclusions

In summary, we have presented a novel FP-CSI framework and demonstrated its application in measuring HAR and composite micro-trenches. FP-CSI integrates the strengths of angular spectrum synthesis and low-coherence interferometry into a unified framework that addresses key limitations of traditional 3D metrology techniques. Its transmissive configuration and angular diversity enable high-precision, non-destructive imaging of HAR microstructures while preserving signal fidelity and eliminating the need for iterative phase retrieval. The effectiveness of FP-CSI was demonstrated by measuring different types of HAR micro-trenches, including a HAR micro-trench (30:1 aspect ratio) and MEMS pressure sensors with composite comb structures (aspect ratios from 6:1 to 20:1). Through careful consideration of dispersion compensation, system calibration, and signal correction, FP-CSI achieves robust reconstruction and a lateral resolution up to the incoherent diffraction limit, validated using a bare resolution target and further sustained when the target was positioned at the bottom of a simulated trench with an aspect ratio >10:1. The method’s adaptability to complex structures and potential for integration with advanced computational techniques further underscore its promise for broad applications in next-generation optical metrology.

## Materials and methods

### Sample fabrication and characterization standards

HAR microstructure samples for experimental validation were fabricated by the 13th Research Institute of China Electronics Technology Group Corporation (Hebei Province, China). The test specimens include standard single-trench structures with widths of 30 μm and 10 μm at depths of 300 μm, fabricated on 550-μm-thick monocrystalline silicon substrates, as well as silicon-based MEMS pressure sensors realized on 600-μm-thick and 620-μm-thick substrates. All samples were produced on monocrystalline silicon substrates using photolithography followed by deep reactive ion etching. Characteristic parameters of the composite HAR micro-trench samples were defined and quantified according to the terminology and definitions specified in ISO/DIS 25178-700:2022^43^. Detailed specifications and SEM images of the fabricated structures are provided in Supplementary Note S3.

### Imaging acquisition and analysis

Image acquisition was automated in MATLAB to synchronize the sensor with the illumination system. To comprehensively sample the specimen’s spatial-frequency components, interferograms were recorded by angular and axial scanning. Angular sampling was performed by scanning a quasi-point source along the *x*- and *y*-directions with a 10 μm step size. One hundred positions were acquired along each axis, resulting in 200 angular views and corresponding sub-apertures in the spatial-frequency domain. For each illumination angle, the reflective mirror in the reference arm and the sample were synchronously translated along the optical axis to maintain alignment with the coherence peak corresponding to different axial layers.

Axial scanning was implemented in two stages to ensure accuracy and efficiency. A coarse scan with a 2 μm step size was first used to localize the coherence envelope peak, corresponding to the surface depth. A refined scan was then performed as described in the “The FP-CSI reconstruction algorithm” section using finer step sizes and 100 increments determined by the coherence length of the broadband source. For each angular illumination, this procedure generated 100 depth-resolved interferograms per surface. The camera exposure time was 16.7 ms, and each translation step required approximately 10 ms, resulting in a total acquisition time of approximately 9 min for all axial and angular scans. The long acquisition time is primarily limited by the extended exposure time required in the near-infrared camera and the mechanical displacement of the light source for angular spectrum scanning. With more advanced hardware, such as cameras with exposure times as short as 1 ms or spatial light modulators for rapid source modulation, the total acquisition time could be reduced to <1 min.

Following the acquisition, the signals were pre-processed to suppress envelope drift and amplitude modulation artifacts, particularly those arising under large-angle illumination. Empirical mode decomposition was applied for correction, after which high-fidelity phase maps were extracted using the centroid method. Each angular phase map corresponded to a circular subregion in Fourier space with a radius of 10 pixels. Adjacent sub-apertures overlapped by ~80 pixels, and averaging was applied to resolve minor inconsistencies. Aperture synthesis and two-dimensional inverse Fourier transform provided the final high-resolution phase map, which was converted to surface height using the standard optical phase-to-height relationship. All processing was implemented in MATLAB R2022a on a Windows workstation (Intel Core i9-13900K, 5.80 GHz; 128 GB RAM). The total time required to reconstruct the image from various interferograms captured under different illumination angles was approximately 8 min. Future implementations are expected to benefit from GPU-based parallelization, which could significantly decrease computation time.

## Supplementary information

Supplementary Information for Fourier ptychographic coherence scanning interferometry for 3D morphology of high aspect ratio and composite micro-trenches.

## Data Availability

All data are available in the main text or the supplementary materials from the corresponding author upon reasonable request.
