# Supplementary material for: Fourier ptychographic coherence scanning interferometry for 3D morphology of high aspect ratio and composite micro-trenches
Source: Light Sci Appl. 2026 Jan 29;15:93. doi: 10.1038/s41377-026-02189-6 (PMC12852827; doi:10.1038/s41377-026-02189-6)
Supplement: Supplementary file 1 — Supplementary Information for Fourier ptychographic coherence scanning interferometry for 3D morphology of high aspect ratio and composite micro-trenches. [file 41377_2026_2189_MOESM1_ESM.pdf]

Supplementary Information for Fourier ptychographic coherence scanning interferometry for 3D morphology of high aspect ratio and composite micro-trenches

Yin Li[1], Qun Yuan[1,], Xiao Huo[1], Shumin Wang[1], Hongtao He[2], and Zhishan Gao[1,*]**

[1]School of Electronic and Optical Engineering, Nanjing University of Science and Technology, Nanjing, Jiangsu Province 210094, China.
[2]The 13th Research Institute of China Electronics Technology Group Corporation, Shijiazhuang, Hebei Province 050052, China.
[*]zhishgao@njust.edu.cn
[**]karmen86913@gmail.com

ABSTRACT

This document provides supplementary information for “Fourier ptychographic coherence scanning interferometry for 3D morphology of high aspect ratio and composite micro-trenches.” It includes the theoretical models, specific reconstruction algorithm, experimental validations, and comparative analysis.

Contents

Supplementary Note S1. Forward model of transmissive coherence scanning interferometry
Supplementary Note S2. Detailed description of the Fourier ptychographic coherence scanning interferometry (FP-CSI) reconstruction algorithm
Supplementary Note S3. Validation of the effectiveness of the FP-CSI method based on experiments
Supplementary Note S4. Consistency analysis and overlap handling in angular spectrum stitching
Supplementary Note S5. Comparison of the FP-CSI method with current state-of-the-art techniques

Supplementary Note S1. Forward model of transmissive coherence scanning interferometry

The optical field generated by a polychromatic, statistically stationary source can be represented as a random process[1]. The analytic signal of the field in arm 1 can be written in the frequency domain as

$$E_1(t) = \frac{1}{2\pi}\int_{-\infty}^{\infty} \tilde{E}_1(\omega)e^{-i\omega t}d\omega, \tag{S1}$$

and the field in arm 2 in the same general form,

$$E_2(t) = \frac{1}{2\pi}\int_{-\infty}^{\infty} \tilde{E}_2(\omega)e^{-i\omega t}d\omega. \tag{S2}$$

Introducing a relative time delay τ between the two arms, the total field at the detector is therefore

$$E(t) = E_1(t) + E_2(t-\tau). \tag{S3}$$

The detected intensity is the ensemble average of the squared modulus of the total field,

$$I = \langle |E(t)|^2 \rangle, \tag{S4}$$

which separates into the individual arm intensities and interference cross-terms,

$$I = I_1 + I_2 + 2\mathrm{Re}\{\Gamma_{12}(\tau)\}, \tag{S5}$$

where $I_j = \langle |E_j(t)|^2 \rangle$ and $\Gamma_{12}(\tau) = \langle E_1(t)E_2^*(t+\tau)\rangle$ is the mutual coherence function. The rigorous description of these terms is provided by the cross-spectral density

$$W_{12}(\omega) = \langle \tilde{E}_1(\omega)\tilde{E}_2^*(\omega)\rangle, \tag{S6}$$

with corresponding auto-spectral densities $W_{11}(\omega)$ and $W_{22}(\omega)$. The Wiener–Khinchin theorem[2] relates the mutual coherence function to the cross-spectral density as

$$\Gamma_{12}(\tau) = \frac{1}{2\pi}\int_{-\infty}^{\infty} W_{12}(\omega)e^{-i\omega\tau}d\omega, \tag{S7}$$

and the intensities of the individual arms as

$$I_j = \frac{1}{2\pi}\int_{-\infty}^{\infty} W_{jj}(\omega)d\omega. \tag{S8}$$

It is convenient to normalize the cross-term by defining the complex degree of coherence

$$\mu_{12}(\tau) = \frac{\Gamma_{12}(\tau)}{\sqrt{I_1 I_2}}, \tag{S9}$$

which allows the detected intensity to be expressed compactly as

$$I(\tau) = I_1 + I_2 + 2\sqrt{I_1 I_2}\mathrm{Re}\{\mu_{12}(\tau)\}. \tag{S10}$$

To obtain an explicit expression for μ_{12}, we model the source spectrum. Since the mutual coherence function is the Fourier transform of the normalized spectrum, it is convenient to change variables to the wavenumber $k = \omega/c$, which is conjugate to the optical path difference (OPD) $\delta = c\tau$. For a Gaussian source spectrum, which is widely used to model low-coherence sources[3], the normalized spectral density is

$$S(k) = \frac{1}{\Delta k\sqrt{\pi}} \exp\left[-\left(\frac{k-k_0}{\Delta k}\right)^2\right], \quad \int_{-\infty}^{\infty} S(k)dk = 1, \tag{S11}$$

where k_0 is the central wavenumber and Δk is the spectral bandwidth, corresponding to the half-width of the spectrum at $1/e$ of its maximum, as illustrated in Fig. S1. The complex degree of coherence then follows as

$$\mu_{12}(\delta) = \int_{-\infty}^{\infty} S(k)e^{-ik\delta}dk = \exp\left[-\frac{(\Delta k\delta)^2}{4}\right] e^{-ik_0\delta}. \tag{S12}$$

In engineering practice, the coherence length l_c is defined as the OPD at which the envelope of the degree of coherence falls to half its maximum[4]. For a Gaussian source, this yields

$$l_c = \frac{2\sqrt{\ln 2}}{\Delta k}, \tag{S13}$$

and thus the degree of coherence may be written as

$$\mu_{12}(\delta) = \exp\left[-\ln 2\left(\frac{\delta}{l_c}\right)^2\right] e^{-ik_0\delta}. \tag{S14}$$

Substituting this into the expression for the intensity gives the low-coherence interference signal,

$$I(\delta) = I_1 + I_2 + 2\sqrt{I_1 I_2}\exp\left[-\ln 2\left(\frac{\delta}{l_c}\right)^2\right]\cos(k_0\delta). \tag{S15}$$

This expression represents the forward model of low-coherence interferometry: the detected signal consists of fringes at carrier frequency k_0, modulated by a Gaussian envelope whose width is determined by the coherence length l_c. In the transmissive architecture of coherence scanning interferometry, the OPD can be written as

$$\delta = (n-n_0)\frac{z}{\cos\theta}, \tag{S16}$$

where z is the sample thickness, θ is the incidence angle, n is the refractive index of the sample, and n_0 is the refractive index of air. Substitution into Eq. (S15) results in the forward model for Fourier ptychographic coherence scanning interferometry (FP-CSI).

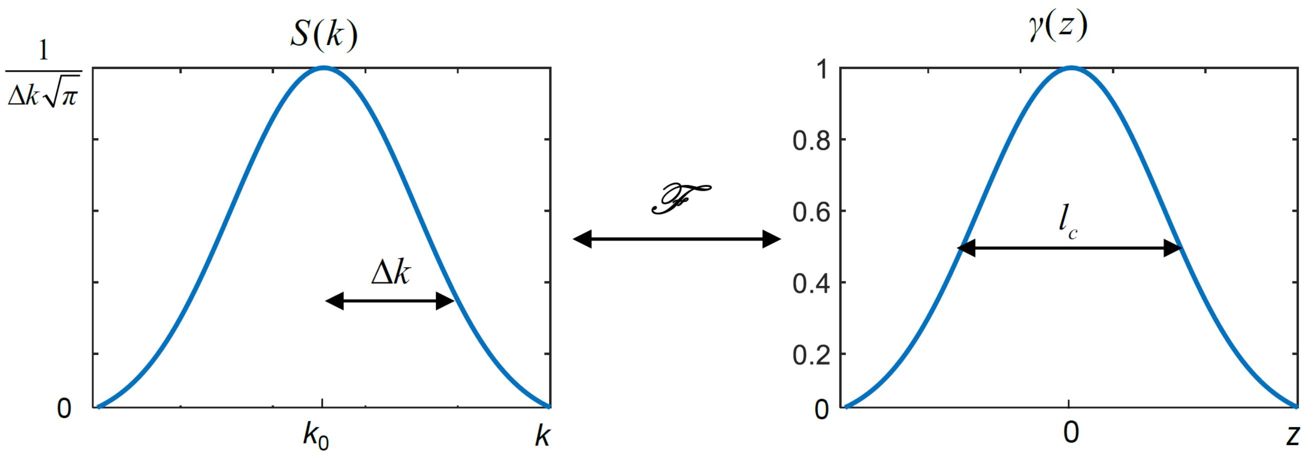

Figure S1. Illustration of Fourier transform relationship between the Gaussian-shaped coherence function $\gamma(z)$ (characterized by the coherence length l_c and the light source spectrum $S(k)$ (characterized by the central wave number k_0 and wave number bandwidth Δk).

Supplementary Note S2. Detailed description of the Fourier ptychographic coherence scanning interferometry (FP-CSI) reconstruction algorithm

Based on the forward model described in the first section of the supplementary materials, we apply the FP-CSI method to obtain the detected interference signal, which encodes both depth and angular information. By scanning over multiple axial positions and illumination angles, a series of depth-resolved interferograms is acquired. This angular diversity enriches the spatial frequency coverage and enables computational synthesis of high-resolution topographic reconstructions. To recover high-resolution 3D morphology from FP-CSI data, we extend the concept of aperture synthesis[5], widely used in Fourier ptychographic microscopy (FPM), to coherence scanning interferometry (CSI). Unlike conventional FPM, which relies on iterative phase retrieval, FP-CSI exploits the intrinsic quantitative phase sensitivity of interferometry, enabling a direct and non-iterative reconstruction. The procedure consists of five main steps.

Step 1: Interferometer initialization.

The process begins with interferometer initialization, where the upper surface of the sample is positioned at the parfocal plane of the test-arm objectives. In addition, a compensation plate is inserted to correct for dispersion, ensuring optical path match and stable interference.

Step 2: Axial and angular scanning to acquire interferograms.

The reflective mirror in the reference arm is translated in increments of $\lambda/8$ to achieve a stepwise optical path difference modulation of $\lambda/4$, where λ denotes the illumination center wavelength. To ensure that the sample plane coincides with the optimal interference position throughout the axial scanning, the

sample is synchronously translated by

$$\Delta z_{\text{sample}} = \frac{1}{4}\lambda \cdot \frac{1}{n - n_0}, \tag{S17}$$

where n and n_0 are the refractive indices of the sample and air, respectively.

Additionally, angular diversity is introduced by shifting the quasi-point source across the illumination plane. The maximum illumination angle is limited by the objective's numerical aperture (NA). Moreover, the pinhole at the pupil plane is co-shifted with the light source to maintain spatial conjugation throughout scanning. The oblique angle θ of the incident light is

$$\theta(m,n) = \arctan \frac{\beta\sqrt{m^2+n^2}}{f}, \tag{S18}$$

where (m,n) is the spatial coordinates of the translating quasi-point light source, β is the magnification ratio from the light source to the objective pupil plane, and f represents the focal length of the objective lens. Therefore, each set of angular and axial scanning positions corresponds to an interferogram.

Step 3: Signal correction using EMD and phase extraction via the centroid method.

Interference signals acquired at large angles are often affected by intensity drift and asymmetrical envelopes due to projection effects. To address this, empirical mode decomposition (EMD)[6] is applied to adaptively remove low-frequency distortions. The centroid method is then used to extract high-fidelity phase maps for each angular view. According to the forward imaging model, the low-coherence interference signal can be expressed as a combined modulation of a Gaussian and a cosine function, symmetric around the zero OPD. By calculating the centroid position of the coherence signal, the zero OPD can be located, and the relative height of each sampling point is obtained. During centroid computation, the DC component of the signal is removed, and the AC component is calculated as

$$\varphi = \frac{\pi}{2} \cdot \frac{\sum i \cdot |I_i'|}{\sum |I_i'|}, \quad I_i' = I_i - \frac{\sum I_i}{N}, \tag{S19}$$

where N is the total number of coherence scanning steps.

However, for low-coherence systems, dispersion can reduce centroid accuracy due to the shifting of different wavelengths within the spectral range. This procedure is equivalent to bringing a DC noise into each light intensity value, introducing asymmetry of the synthesizing signal, and thus reducing the calculation accuracy of the centroid method. To overcome this, Ai et al.[7] proposed an improved centroid method, replacing intensity values with squared differences between adjacent samples:

$$\varphi = \frac{\pi}{2} \cdot \frac{\sum i \cdot d_i}{\sum d_i}, \quad d_i = [I_{i+1} - I_i]^2. \tag{S20}$$

This effectively filters out dispersion-induced bias and improves height measurement accuracy. In addition, FP-CSI performs coherence scanning with a fixed step size corresponding to a phase shift of $\pi/2$, enabling phase and contrast extraction using the Sandoz seven-frame method[8]:

$$\varphi = \arctan\left(\frac{3I_3 + I_7 - I_1 - 3I_5}{4I_4 - 2I_2 - 2I_6}\right), \tag{S21}$$

$$M = \begin{cases} \dfrac{3I_3 + I_7 - I_1 - 3I_5}{\sin\varphi}, & \sin\varphi \geq \cos\varphi, \\ \dfrac{4I_4 - 2I_2 - 2I_6}{\cos\varphi}, & \sin\varphi < \cos\varphi. \end{cases} \tag{S22}$$

Step 4: Angular spectrum stitching in the Fourier domain.

Each angularly resolved phase map $\varphi_{(m,n)}(x,y)$ at object coordinates (x,y) is associated with a specific subregion of the spatial frequency domain. The effective frequency component for each view is given by

$$O_{(m,n)}(u,v) = \mathscr{F}\{\varphi_{(m,n)}(x,y)\} \cdot P_{(m,n)}(u,v), \tag{S23}$$

$$P_{(m,n)}(u,v) = \begin{cases} 1, & (u-\beta m)^2 + (v-\beta n)^2 \leq r^2, \\ 0, & (u-\beta m)^2 + (v-\beta n)^2 > r^2, \end{cases} \tag{S24}$$

where $\mathscr{F}$ denotes the Fourier transform, $P_{(m,n)}(u,v)$ denotes the pupil function at pupil coordinates (u,v) and r is the light source image in the objective pupil plane. This radius sets the effective cutoff frequency, suppressing high-frequency diffraction noise. All subregions are linearly combined in Fourier space:

$$\varphi(x,y) = \mathscr{F}^{-1}\left\{\sum_m \sum_n O_{(m,n)}(u,v)\right\}. \tag{S25}$$

No iterative refinement is necessary due to the overlap between adjacent subregions. This overlap significantly simplifies the reconstruction process and enhances computational efficiency.

Step 5: Conversion of the stitched phase into 3D surface topography.

The final stitched phase map can be transformed into surface height according to Eq. (S26), thus achieving a high-resolution 3D topography of the sample.

$$h(x,y) = \frac{\lambda}{2\pi}\varphi(x,y). \tag{S26}$$

Supplementary Note S3. Validation of the effectiveness of the FP-CSI method based on experiments

For trench structures, ISO: 25178-700[9] defines characteristic parameters including width W and depth d (Fig. S2a). To avoid the influence of any rounding of the corners, the upper surface on each side of the trench is to be ignored for a length equal to one-third of its width. The surface at the bottom of the trench is assessed only over the central third of its width. The portions to be used for assessment purposes are those shown at A, B, and C in Fig. S2b. To quantitatively analyze the experimental data, the standard deviation is calculated as

$$s = \sqrt{\frac{1}{n-1}\sum_{i=1}^{n}(x_i - \bar{x})^2}, \tag{S27}$$

where n is the number of samples, x_i is the $i-$th measured value, and $\bar{x}$ is the mean value. The repeatability s_r and the relative error δ_{rel} are given by

$$s_r = \frac{s}{x_{SEM}} \times 100\%, \quad \delta_{rel} = \frac{|\bar{x} - x_{SEM}|}{x_{SEM}} \times 100\%, \tag{S28}$$

where x_{SEM} represents the measurement result obtained by the scanning electron microscopy (SEM).

To validate the effectiveness of FP-CSI, multiple HAR micro-trench samples were measured. Each sample was measured 10 times to ensure statistical reliability. Fig. S2c depicts the SEM image of a micro-trench with a 29.8 μm linewidth and 300.07 μm depth (aspect ratio 10:1), while Fig. S2d shows an SEM image of a micro-trench with a 9.98 μm linewidth and 302.96 μm depth (aspect ratio 30:1). The reconstructed parameters extracted from FP-CSI are summarized in Tables S1 and S2, respectively. Across both cases, FP-CSI achieved measurement relative errors below 1.00% and repeatability better than 0.95%, confirming its robustness for HAR micro-trench metrology.

We further evaluated FP-CSI on multilayer micro-electro-mechanical systems (MEMS) pressure sensors to assess its capability in recovering the morphology of composite HAR devices. Figs. S2e and S2f show SEM images of two representative sensors with comb-like trench structures. Sensor 1 consists of two trenches with nominal dimensions: 10.57 μm linewidth and 106.98 μm depth (aspect ratio 10:1), and 21.15 μm linewidth and 124.41 μm depth (aspect ratio 6:1). Sensor 2 consists of two trenches with dimensions of 4.55 μm linewidth and 89.71 μm depth (aspect ratio 20:1) and 13.7 μm linewidth and 117.57 μm depth (aspect ratio 9:1). The reconstructed parameters are summarized in Tables S3 and S4. For both sensors, FP-CSI achieved relative errors and repeatability better than 0.97%, demonstrating its effectiveness in characterizing multilayer and composite HAR devices.

These results collectively confirm the accuracy, repeatability, and robustness of FP-CSI for quantitative measurement of both isolated and composite HAR microstructures. Beyond these demonstrations, FP-CSI holds promise for broader applications, including failure analysis in semiconductor devices, in situ monitoring of MEMS fabrication, inspection of three-dimensional integrated circuits, and volumetric imaging of biological tissues encapsulated in scattering media. These potential extensions highlight FP-CSI as a versatile platform for high-resolution, non-destructive characterization of complex microstructures.

Table S1. Reconstructed parameters of a 10:1 aspect ratio micro-trench

Parameter	Width	Depth
Measurement value / μm	29.92	301.76
	30.02	303.04
	30.03	298.88
	29.77	300.03
	29.84	301.33
	29.76	298.57
	30.31	304.44
	30.16	302.19
	29.88	306.58
	30.17	299.34
Mean value / μm	29.99	301.62
Standard deviation / μm	0.18	2.57
Repeatability	0.62%	0.86%
Relative error	0.62%	0.52%

Table S2. Reconstructed parameters of a 30:1 aspect ratio micro-trench

Parameter	Width	Depth
Measurement value / μm	9.96	302.56
	10.05	304.52
	10.11	304.35
	9.91	300.00
	10.09	301.05
	10.12	303.11
	10.19	299.75
	10.18	300.41
	10.02	302.97
	10.17	303.49
Mean value / μm	10.08	302.22
Standard deviation / μm	0.09	1.78
Repeatability	0.95%	0.59%
Relative error	1.00%	0.24%

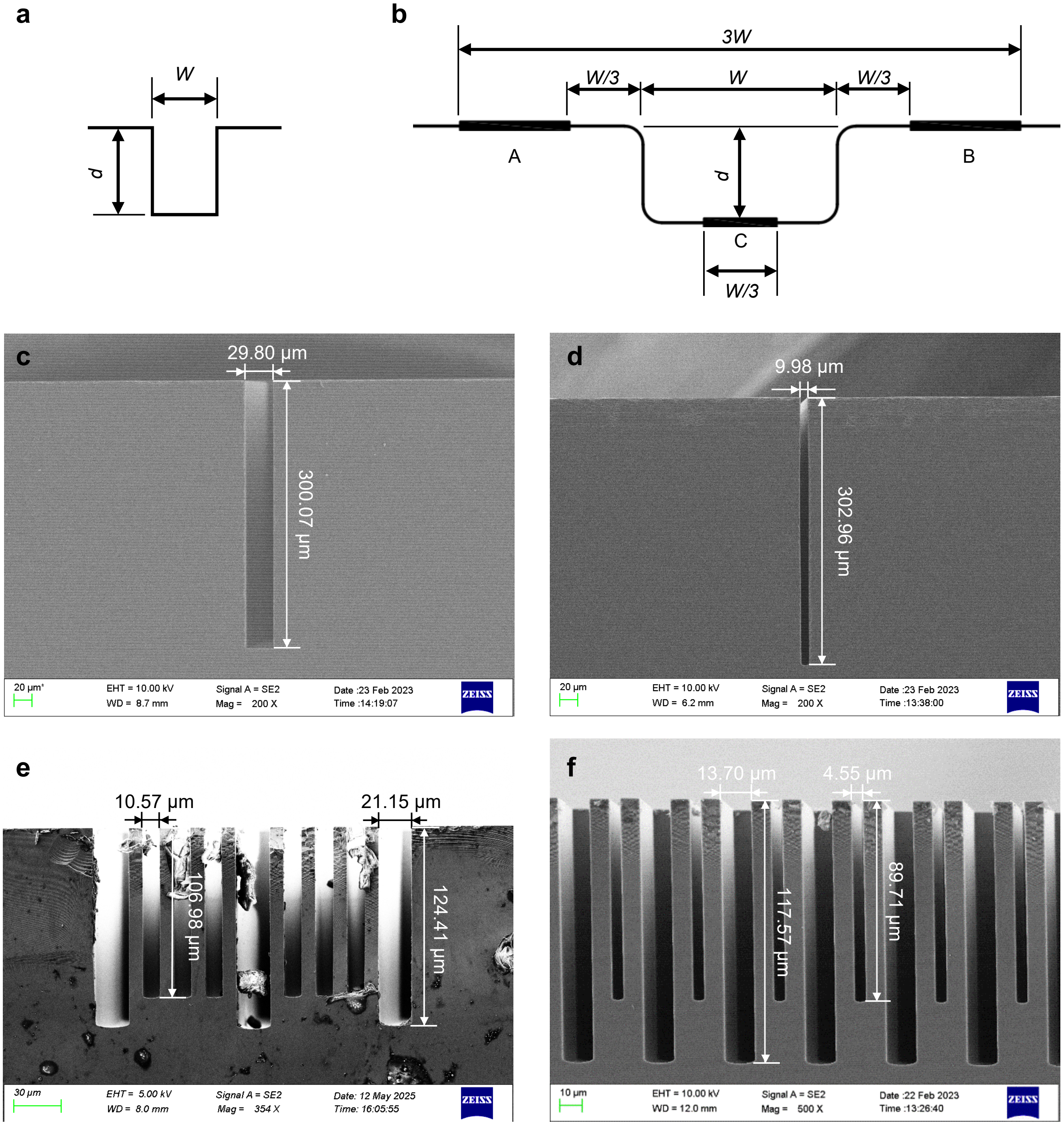

Figure S2. Definition of trench parameters and SEM images of test samples. a Standard rectangular trench. **b** ISO 25178-70 assessment regions for trench width and depth. **c, d** SEM images of HAR micro-trenches with aspect ratios of 10:1 and 30:1, respectively. **e, f** SEM images of two MEMS pressure sensors with composite comb structures.

Table S3. Reconstructed parameters of MEMS pressure sensor 1

Parameter	Width 1	Depth 1	Width 2	Depth 2
Measurement value / μm	10.62	106.71	21.36	125.33
	10.74	107.35	21.00	124.91
	10.73	106.70	21.12	125.72
	10.59	106.96	21.48	125.37
	10.54	107.65	21.37	123.33
	10.71	108.47	21.44	124.83
	10.58	108.01	21.51	123.34
	10.73	107.63	21.25	125.32
	10.66	106.34	21.13	123.50
	10.55	107.65	21.04	125.27
Mean value / μm	10.64	106.98	21.27	124.69
Standard deviation / μm	0.08	0.66	0.19	0.93
Repeatability	0.75%	0.62%	0.89%	0.75%
Relative error	0.71%	0.34%	0.57%	0.23%

Table S4. Reconstructed parameters of MEMS pressure sensor 2

Parameter	Width 1	Depth 1	Width 2	Depth 2
Measurement value / μm	4.57	90.53	13.82	117.74
	4.59	91.37	13.75	116.91
	4.57	89.56	13.59	119.14
	4.55	90.11	13.62	118.90
	4.65	91.04	13.91	118.36
	4.53	91.54	13.64	117.35
	4.52	88.95	13.70	118.04
	4.58	90.34	13.70	118.74
	4.59	89.33	13.93	118.71
	4.57	89.79	13.82	117.40
Mean value / μm	4.57	90.26	13.75	118.13
Standard deviation / μm	0.04	0.87	0.12	0.76
Repeatability	0.79%	0.97%	0.87%	0.64%
Relative error	0.48%	0.61%	0.35%	0.48%

Supplementary Note S4. Consistency analysis and overlap handling in angular spectrum stitching

Low-coherence scanning interferometry (CSI) provides direct access to the quantitative phase of a test wavefront, but its lateral resolution is restricted by the objective NA. For HAR microstructures, a large NA introduces significant modulation, which reduces interference contrast and prevents phase demodulation. Conventional FPM bypasses this limitation by synthesizing a large NA from variable tilted illuminations, yet it relies on iterative phase retrieval that suffers from angle mismatch and error accumulation. To resolve these issues, FP-CSI integrates the complementary strengths of CSI and FPM. In FP-CSI, the wavefront phase under angularly scanned illumination is directly measured by CSI, and these phase-resolved datasets are assembled in the Fourier domain, avoiding iterative reconstruction errors. Overlaps between angular subregions are intentionally designed to ensure the spectral fidelity.

The consistency of overlapping regions was examined using the HAR micro-trench sample (30 μm width, 300 μm depth, 10:1 aspect ratio) described in the main text. Phase maps from three neighboring acquisitions along the x-axis (Figs. S3a1–a3) and y-axis (Figs. S4a1–a3) were Fourier transformed to obtain their frequency spectra (Figs. S3b1–b3 and S4b1–b3). The modulus of spectral differences between adjacent acquisitions (Figs. S3c1, c2 and S4c1, c2) shows that deviations in overlapping regions are significantly smaller than in non-overlapping regions, demonstrating that FP-CSI achieves highly consistent phase recovery across illumination angles and allows direct aperture stitching of complex amplitudes.

To further minimize residual mismatches, we employed an averaging strategy in which overlapping regions were replaced by the mean of corresponding complex amplitude values. This approach produced smooth boundary transitions, maintained high-frequency phase information, and proved robust across multiple experimental datasets. In summary, the overlap analysis confirms the internal consistency of angular phase recovery in FP-CSI and validates the averaging scheme as an effective stitching strategy, ensuring reliable high-fidelity reconstructions of HAR microstructures.

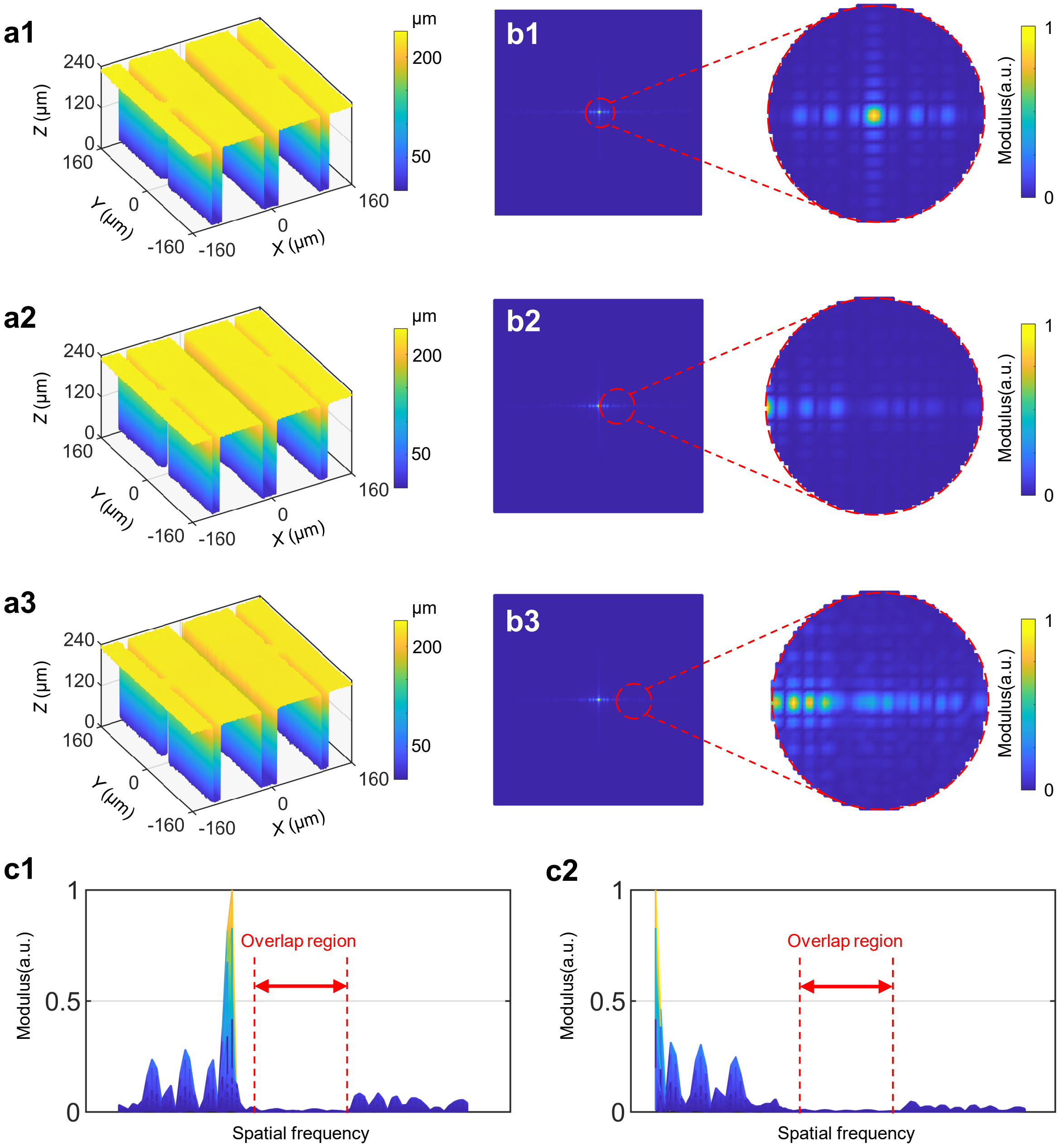

Figure S3. **Consistency analysis of angular acquisitions along the *x*-axis. a1–a3** Phase images from three adjacent illuminations. **b1–b3** Corresponding Fourier spectra. **c1, c2** Modulus of spectral differences between neighboring spectra.

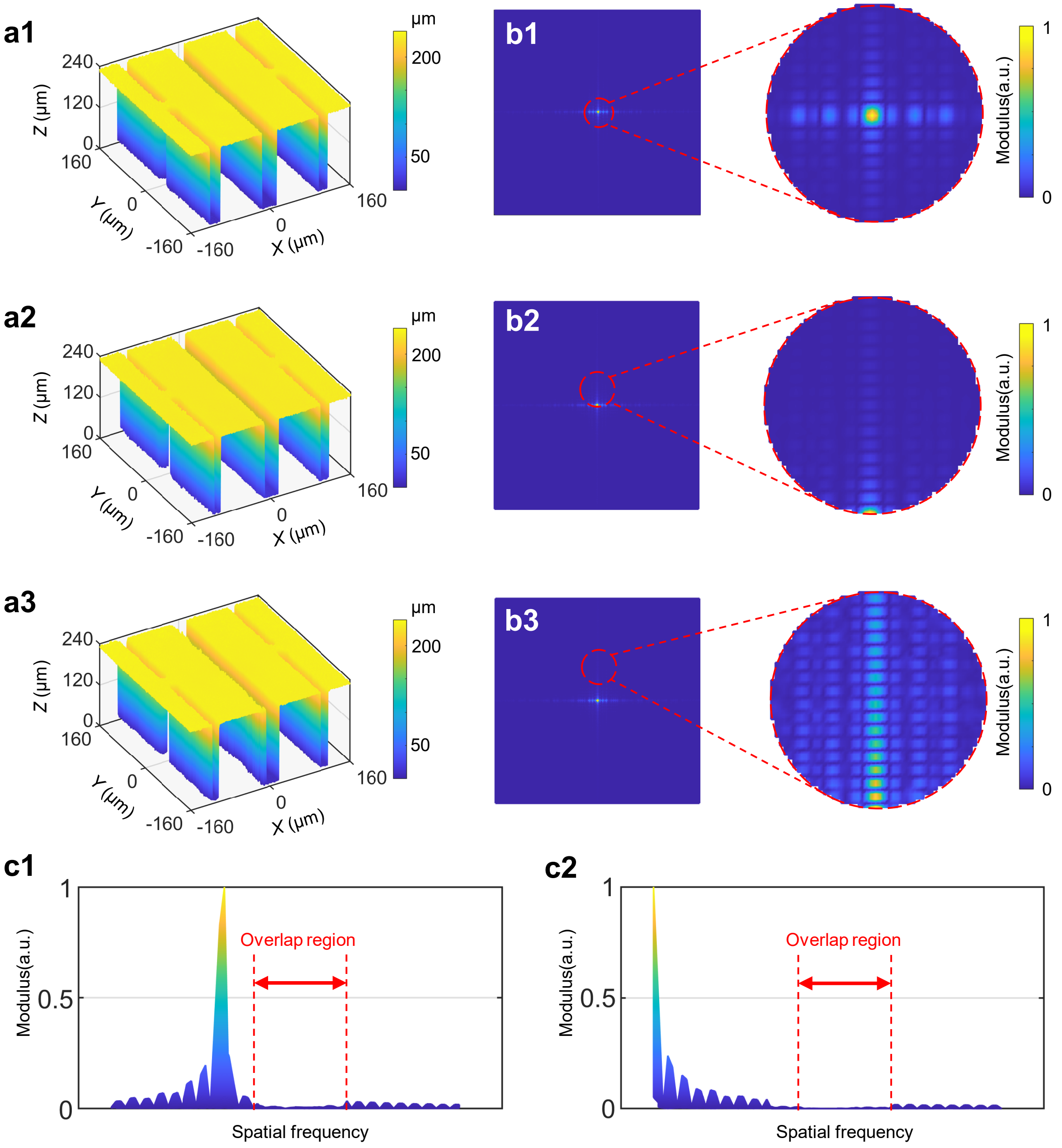

Figure S4. Consistency analysis of angular acquisitions along the *y*-axis. a1–a3 Phase images from three adjacent illuminations. **b1–b3** Corresponding Fourier spectra. **c1, c2** Modulus of spectral differences between neighboring spectra.

Supplementary Note S5. Comparison of the FP-CSI method with current state-of-the-art techniques

Table S5 summarizes representative metrology techniques that have been used for the characterization of HAR microstructures. For each method, the table lists the main measurement output (i.e., full topography versus statistical parameters), the maximum measurable aspect ratio, the typical acquisition time, and the dominant limitation. The abbreviations follow the caption of Table S5.

Table S5. Comparison of existing optical metrology techniques for HAR microstructures. Max AR: maximum measurable aspect ratio; AFM: atomic force microscopy; SEM: scanning electron microscopy; CT: computed tomography; UT: ultrasonic testing; TSOM: through-focus scanning optical microscopy; SR: spectroscopic reflectometry; OS: optical scatterometry; OCT: optical coherence tomography; CSI: coherence scanning interferometry; FP-CSI: Fourier ptychographic coherence scanning interferometry; SNR: signal-to-noise ratio.

Methodology	Output	Max AR	Time	Limitation
AFM	Topography	$>$100:1	15-30 min	Destructive
SEM	Topography	$>$100:1	5-10 min	Destructive
CT	Topography	$\sim$5:1	1-2 hour	Long acquisition time
UT	Topography	$\sim$2:1	30-60 min	Low resolution
TSOM	Parameters	$\sim$85:1	5-10 s	Sample library requirement
SR	Parameters	$\sim$5:1	5-10 s	Sensitive to roughness
OS	Parameters	$\sim$10:1	5-10 s	Sensitive to non-periodicity
OCT	Topography	$\sim$10:1	5-10 s	Low axial resolution
CSI	Topography	$\sim$20:1	1-3 min	SNR-to-resolution trade-off
FP-CSI	Topography	$\sim$30:1	9-15 min	Transmissive sample

S5.1 Overview of existing techniques

Destructive techniques

Atomic force microscopy (AFM) and scanning electron microscopy (SEM) provide direct topographic information with nanometer-scale accuracy and can access enormous aspect ratios. However, both techniques are destructive because cross-sectioning or sample preparation is required for HAR microstructures. The associated acquisition time is relatively long, which restricts their use to sampling-based inspection rather than in-line or full-field metrology. For instance, Colston et al.[10] characterized micro-trenches with

a width of 2 μm and a depth of 5 μm using AFM. Additionally, Atak et al.[11] observed HAR metastructure resonator images with widths ranging from 2 to 3 μm and depths between 10 and 11 μm using SEM. These two destructive measurement methods are commonly employed because they can achieve precision at the nanometer level. While these methods have certain limitations, they serve as valuable references and standards for non-destructive measurement techniques.

Non-destructive volumetric techniques

Computed tomography (CT) and ultrasonic testing (UT) are both non-destructive volumetric techniques. For example, Jing et al.[12] employed commercial X-ray microscopy tools to visualize metallization defects in vias (aspect ratio 5:1). Kim et al.[13] utilized an acoustic microscopy system to identify internal defects in through-silicon vias (TSVs) with 30 μm width and 60 μm depth. According to Table S5, CT has a maximum measurable aspect ratio of approximately 5:1 and typically requires an acquisition time of 1 to 2 hours. Its primary drawback is this lengthy acquisition time. Additionally, UT has a smaller maximum measurable aspect ratio of around 2:1 and takes 30 to 60 minutes per measurement, but it is inherently limited by low spatial resolution. These factors render both CT and UT unsuitable for high-throughput dimensional metrology of HAR micro-features.

Optical parameter-retrieval techniques

Through-focus scanning optical microscopy (TSOM), spectroscopic reflectometry (SR), and optical scatterometry (OS) are non-destructive optical techniques that infer structure parameters by comparing measured signals with precomputed libraries or models. Consistent with this, Table S5 classifies their output as parameters rather than full topography. TSOM can handle enormous aspect ratios (maximum measurable aspect ratio $\sim$85:1) with short acquisition times of 5–10 s but relies on an extensive sample library. SR achieves a maximum measurable aspect ratio of $\sim$35:1 within 5–10 s, yet is sensitive to surface roughness. OS exhibits a maximum measurable aspect ratio of $\sim$10:1 with 5–10 s acquisition time, but its performance is strongly affected by deviations from periodicity. Consequently, these methods are well suited for statistical process control of nominally periodic structures, but they do not provide detailed 3D morphology for arbitrary HAR microstructures.

For instance, Li et al.[14] proposed a TSOM method that integrates a convolutional neural network model for statistically measuring individual HAR micro-trenches, achieving an aspect ratio of 85:1. Bauer et al.[15] developed a scatterometry-based setup for determining the depths of very deep silicon vias, with an aspect ratio of 35:1. Additionally, Chein et al.[16] utilized non-integral model-based scatterometry to measure RDL structures, which have an aspect ratio of 3:1. However, these model-based inversion methods generally provide only statistical data and lack detailed morphological insights, which restricts their applicability for advanced inspection.

Optical morphology-retrieval techniques

Optical coherence tomography (OCT) and coherence scanning interferometry (CSI) directly recover the 3D topography. OCT offers rapid acquisition and a maximum measurable aspect ratio of $\sim$10:1, but its axial resolution is limited[17]. Conventional CSI can reach a maximum measurable aspect ratio of $\sim$20:1 with acquisition times of 1–3 min, yet it suffers from an inherent SNR-to-resolution trade-off[18]. For HAR features, high-NA illumination interacting with deep or steep profiles leads to multiple internal scattering,

strong edge diffraction, and complex wavefront distortions, which markedly reduce the interference contrast and SNR. Recent developments, such as aberration-compensated CSI with a deformable mirror[19] and deconvolution-based CSI using a 3D point-spread-function model[20], partially mitigate these issues. Nevertheless, their demodulation capabilities remain limited, and the practically measurable aspect ratio is still restricted, as reflected by the maximum measurable aspect ratio of ∼20:1.

S5.2 Comparative benchmark of FP-CSI relative to SEM and CSI

FP-CSI is a non-destructive technique that combines aperture synthesis in Fourier ptychography with quantitative phase measurement in a transmissive Linnik interferometer configuration. By varying the illumination angle and synthesizing a high-NA phase map from a series of low-NA acquisitions, FP-CSI is specifically designed to overcome the SNR-to-resolution trade-off that limits conventional CSI in HAR measurements.

As summarized in Table S5, FP-CSI provides full topography with a maximum measurable aspect ratio of ∼30:1 and an acquisition time of 9–15 min. Among the non-destructive methods capable of reconstructing 3D morphology, FP-CSI supports the highest aspect ratio while maintaining quantitative reconstruction. Additionally, the primary constraint of FP-CSI is that the sample must be transmissive, which is explicitly indicated in Table S5.

In the main text, SEM measurements are employed as a reference standard to validate the quantitative accuracy of FP-CSI. For representative HAR trenches and composite MEMS devices, the deviation and repeatability of FP-CSI measurements are within 1% relative to SEM, following the procedures described in Supplementary Note S4. This demonstrates that FP-CSI achieves SEM-comparable accuracy while avoiding destructive sample preparation.

The main manuscript also compares FP-CSI with a state-of-the-art reflective Linnik CSI implementation. For a 30 µm-wide, 300 µm-deep micro-trench, FP-CSI provides higher SNR at the trench bottom than reflective CSI, even when adaptive optics is used to correct sample-induced aberrations. Furthermore, a quantitative assessment of the lateral resolution at the bottom of HAR micro-trenches shows that FP-CSI preserves high-resolution imaging under conditions where reflective CSI is significantly degraded. This improved performance arises from the angular spectrum synthesis in the transmissive configuration, which effectively addresses high-resolution imaging under the interference contrast limitations that constrain conventional CSI.

In summary, Table S5 and the associated comparisons show that FP-CSI bridges the gap between destructive, high-accuracy methods and non-destructive optical metrology. It enables high-resolution 3D reconstruction of HAR microstructures with aspect ratios up to ∼30:1, as well as composite MEMS devices, thereby alleviating the measurement limitations faced by existing morphology-based metrology techniques.

References

1. Goodman, J. W. *Statistical Optics* (John Wiley & Sons, 2015).
2. Ryabukho, V. P., Lyakin, D. V., Grebenyuk, A. A. & et al. Wiener–khintchin theorem for spatial coherence of optical wave field. *J. Opt.* **15**, 025405 (2013).
3. Huang, D., Swanson, E. A., Lin, C. P. & et al. Optical coherence tomography. *Sci.* **254**, 1178–1181 (1991).
4. Born, M. & Wolf, E. *Principles of Optics: Electromagnetic Theory of Propagation, Interference and Diffraction of Light* (Elsevier, 2013).
5. Zheng, G., Horstmeyer, R. & Yang, C. Wide-field, high-resolution fourier ptychographic microscopy. *Nat. Photonics* **7**, 739–745 (2013).
6. Lin, L. & Hongbing, J. Signal feature extraction based on an improved emd method. *Meas.* **42**, 796–803 (2009).
7. Ai, C. & Novak, E. L. Centroid approach for estimating modulation peak in broad-bandwidth interferometry (1997).
8. Sandoz, P. Wavelet transform as a processing tool in white-light interferometry. *Opt. Lett.* **22**, 1065–1067 (1997).
9. International Organization for Standardization. *Geometrical product specifications (GPS) – Surface texture: Areal – Part 700: Calibration, adjustment and verification of areal topography measuring instruments (ISO 25178-700:2020)* (ISO, Geneva, 2020).
10. Colston, G. *et al.* Epitaxial trench refill of 4h-sic by chlorinated chemistry. *Appl. Phys. Lett.* **124** (2024).
11. Atak, A. Ç., Ünal, E. & Demir, H. V. Micro-3d sculptured metastructures with deep trenches for sub-10 μm resolution. *Microsystems & Nanoeng.* **11**, 47 (2025).
12. Jing, X., Yu, D., Wang, W., Yu, G. & Wan, L. Non-destructive testing of through silicon vias by using x-ray microscopy. In *2012 13th International Conference on Electronic Packaging Technology & High Density Packaging*, 1254–1257 (IEEE, 2012).
13. Kim, T. H., Kang, D., Kim, J. N. & Park, I. K. Through-silicon via device non-destructive defect evaluation using ultra-high-resolution acoustic microscopy system. *Mater.* **16**, 860 (2023).
14. Li, G. *et al.* Mems high aspect ratio trench three-dimensional measurement using through-focus scanning optical microscopy and deep learning method. *Appl. Sci.* **12**, 8396 (2022).
15. Bauer, J. *et al.* Very high aspect ratio through silicon via reflectometry. In *Optical Measurement Systems for Industrial Inspection X*, vol. 10329, 872–879 (SPIE, 2017).
16. Chein, W.-H., Yang, F.-S., Thakur, K., Wu, G.-W. & Chen, L.-C. Non-integral depth measurement of high-aspect-ratio multi-layer microstructures using numerical-aperture shaped beams. *Opt. Lasers Eng.* **166**, 107563 (2023).

17. Iff, W. *et al.* Electromagnetic analysis for optical coherence tomography based through silicon vias metrology. *Appl. optics* **58**, 7472–7488 (2019).

18. Jo, T., Kim, S. & Pahk, H. 3d measurement of tsvs using low numerical aperture white-light scanning interferometry. *J. Opt. Soc. Korea* **17**, 317–322 (2013).

19. Ma, J. *et al.* Three-dimensional topography of high-aspect ratio trenches by sample-induced aberration-compensable coherence scanning interferometry. *ACS Photonics* **11**, 1068–1077 (2024).

20. Qiao, W. *et al.* Research on the modulation aberration numerical correction method of interference signals for high aspect ratio samples utilizing coherence scanning interferometry. *Opt. Express* **33**, 330–343 (2025).
